# Assessing Pictograph Recognition: A Comparison of Crowdsourcing and Traditional Survey Approaches

**DOI:** 10.2196/jmir.4582

**Published:** 2015-12-17

**Authors:** Jinqiu Kuang, Lauren Argo, Greg Stoddard, Bruce E Bray, Qing Zeng-Treitler

**Affiliations:** ^1^ Department of Biomedical Informatics University of Utah Salt Lake City, UT United States; ^2^ Study Design and Biostatistics Center University of Utah Salt Lake City, UT United States; ^3^ George E. Wahlen Department of Veterans Affairs Medical Center Informatics Decision-Enhancement and Analytic Sciences (IDEAS) Center Salt Lake City, UT United States

**Keywords:** crowdsourcing, patient discharge summaries, Amazon Mechanical Turk, pictograph recognition, cardiovascular

## Abstract

**Background:**

Compared to traditional methods of participant recruitment, online crowdsourcing platforms provide a fast and low-cost alternative. Amazon Mechanical Turk (MTurk) is a large and well-known crowdsourcing service. It has developed into the leading platform for crowdsourcing recruitment.

**Objective:**

To explore the application of online crowdsourcing for health informatics research, specifically the testing of medical pictographs.

**Methods:**

A set of pictographs created for cardiovascular hospital discharge instructions was tested for recognition. This set of illustrations (n=486) was first tested through an in-person survey in a hospital setting (n=150) and then using online MTurk participants (n=150). We analyzed these survey results to determine their comparability.

**Results:**

Both the demographics and the pictograph recognition rates of online participants were different from those of the in-person participants. In the multivariable linear regression model comparing the 2 groups, the MTurk group scored significantly higher than the hospital sample after adjusting for potential demographic characteristics (adjusted mean difference 0.18, 95% CI 0.08-0.28, *P*<.001). The adjusted mean ratings were 2.95 (95% CI 2.89-3.02) for the in-person hospital sample and 3.14 (95% CI 3.07-3.20) for the online MTurk sample on a 4-point Likert scale (1=totally incorrect, 4=totally correct).

**Conclusions:**

The findings suggest that crowdsourcing is a viable complement to traditional in-person surveys, but it cannot replace them.

## Introduction

Crowdsourcing has become increasingly popular in the past decade due to its time-saving and cost-effective qualities [1,2]. Crowdsourcing was primarily used by industries to outsource business tasks. More recently, human subject researchers have taken interest in crowdsourcing as a viable alternative approach to traditional methods of participant recruitment. The study domains include, but are not limited to, social behavioral science [3], psychology [4-6], and other health-related sciences [7-14]. Crowdsourcing has also been used to generate annotation gold standards for natural language processing in a variety of technical fields [15-22].

In the biomedical domain, researchers have begun experimenting with crowdsourcing. A recent systematic review of crowdsourcing used for health and medical research argued that utilizing crowdsourcing could improve the quality, cost, and speed of a research project and contributes to novel scientific findings [7]. Leroy et al [8] recruited Amazon Mechanical Turk (MTurk) workers to evaluate the effects of a text simplification algorithm using term familiarity to improve perceived and actual text difficulty. Yu et al [9] also crowdsourced a pictogram evaluation task to MTurk workers and confirmed that crowdsourcing can be used as an effective and inexpensive approach for participatory evaluation of medical pictograms.

MTurk is a large and well-known crowdsourcing service, which has developed into the leading platform for crowdsourcing recruitment [23]. Two primary concerns in the use of MTurk for human subject research are the demographic mix of the study participants and data quality, both of which affect the validity and generalizability of results obtained from MTurk. Demographic composition of the participants is essential to understanding the sampling bias of the study population and the generalizability of results. Early studies indicated that workers reached by MTurk were mostly US based and this population was younger, better educated, and with a higher proportion of females than the general US population [24-26]. A 2010 paper by Eriksson and Simpson [26] reported that a greater proportion of Indian participants were recruited (with 424 respondents from India and 416 from the United States) for their experiment. A demographic survey conducted by Paolacci et al [25] showed that only 47% of workers were from the United States and there were a significant number of Indian participants (34%). However, it is rumored that Amazon stopped approving new international MTurk accounts since early 2013.

Regarding data quality, researchers have attempted to understand the motivations of the MTurk participants and whether it affected data quality [17,27-31]. Given that the median wage of MTurk workers was as low as US $1.38 per hour [32], one may be concerned about the quality of work. However, a number of prior studies have compared the quality of data between MTurk workers and in-laboratory participants in various research studies and suggested that the data collected online were not of poorer quality than data collected from traditional subject pools [1-5,25,33].

Specific to the biomedical domain, concerns associated with the use of crowdsourcing include exclusion of certain populations, such as minors and people with limited or no computer skills [2]. Other concerns are built-in limitations that include (1) sample biases, (2) inability to control participants’ environment, and (3) inability to verify participant responses [34]. For researchers who are interested in clinical populations, the prevalence of clinical conditions and clinical characteristics of MTurk workers and the general population may be different. Another issue is that online informed consent documentation is not always read carefully [35]. Despite these concerns, crowdsourcing is a potential alternative to more traditional methods of subject recruitment.

We have been working on improving hospital discharge instructions with automated pictographic illustrations. Hospital discharge instructions are essential to the patients’ postdischarge care because these patients and their families are usually responsible for the majority of care after discharge. However, discharge instructions can be difficult for some patients to understand. Previous studies have shown that more than half of patients do not fully understand the content of instructions [36-38]. Illustrations can help enhance patients’ comprehension and recall [39-41]. However, not all illustrations lead to better comprehension and recall [39,42,43]. Therefore, high-quality and effective pictographs are needed. We created a set of pictographs and stored them in a system called “Glyph.” Glyph automatically illustrates text with analogous pictographs using natural language processing and computer graphics techniques [44]. For Glyph to be effective, we needed to test and ensure that the pictographs it uses are indeed recognizable by patients.

Given crowdsourcing’s low cost, high efficiency, and relatively good data quality, we set out to explore its use for clinical pictograph testing and compare it with a traditional recruitment and survey method. We noted that prior clinical informatics studies have not compared the results obtained from traditional subject recruitment and crowdsourcing. In this study, we tested medical pictograph recognition rate using a hospital sample and using an MTurk sample. MTurk was chosen for this study because it is the most well-established and well-studied crowdsourcing service. It also allowed us to closely control participation and measure the quality of participant output.

## Methods

As part of the Gylph project, more than 1000 pictographs were developed. Among them, we randomly selected 500 pictographs for testing. These pictographs were first drawn by a professional graphic designer and then reviewed by a team of clinicians and researchers. Field testing with patients/consumers was then performed because the patient/consumer population is very diverse and the developers were inherently biased by their participation in the design. To test pictograph recognition, we designed a set of questionnaires with fill-in-the-blank questions for which study participants were asked to complete discharge instruction sentences based on the pictures shown. A total of 150 different questionnaires were generated, each containing 50 questions, enabling each pictograph to be tested 15 times.

After the University of Utah Institutional Review Board approved the in-person survey study, 100 study participants were recruited from a cafeteria area of the University of Utah Hospital, which is frequented by patients, visitors, and staff. Another 50 study participants were recruited from the Environmental Services Department via convenience sampling. Inclusion criteria for participants included individuals aged 21 years or older and able to speak, read, and write in English. Exclusion criteria included anyone unable to read; having any visual, cognitive, language, or other impairments that would prevent full participation in the study; and anyone who currently or previously worked with discharge instructions in any capacity. Informed consent was obtained from each participant. Once consented, participants were given a randomly selected questionnaire, asked to read the questions, fill in the blanks based on the pictographs, and provide their demographic information including age, gender, race, ethnicity, education level, and first language. Most participants completed the questionnaire in 10-20 minutes. Each participant received a US $10 gift card for participation [45].

**Figure 1:**
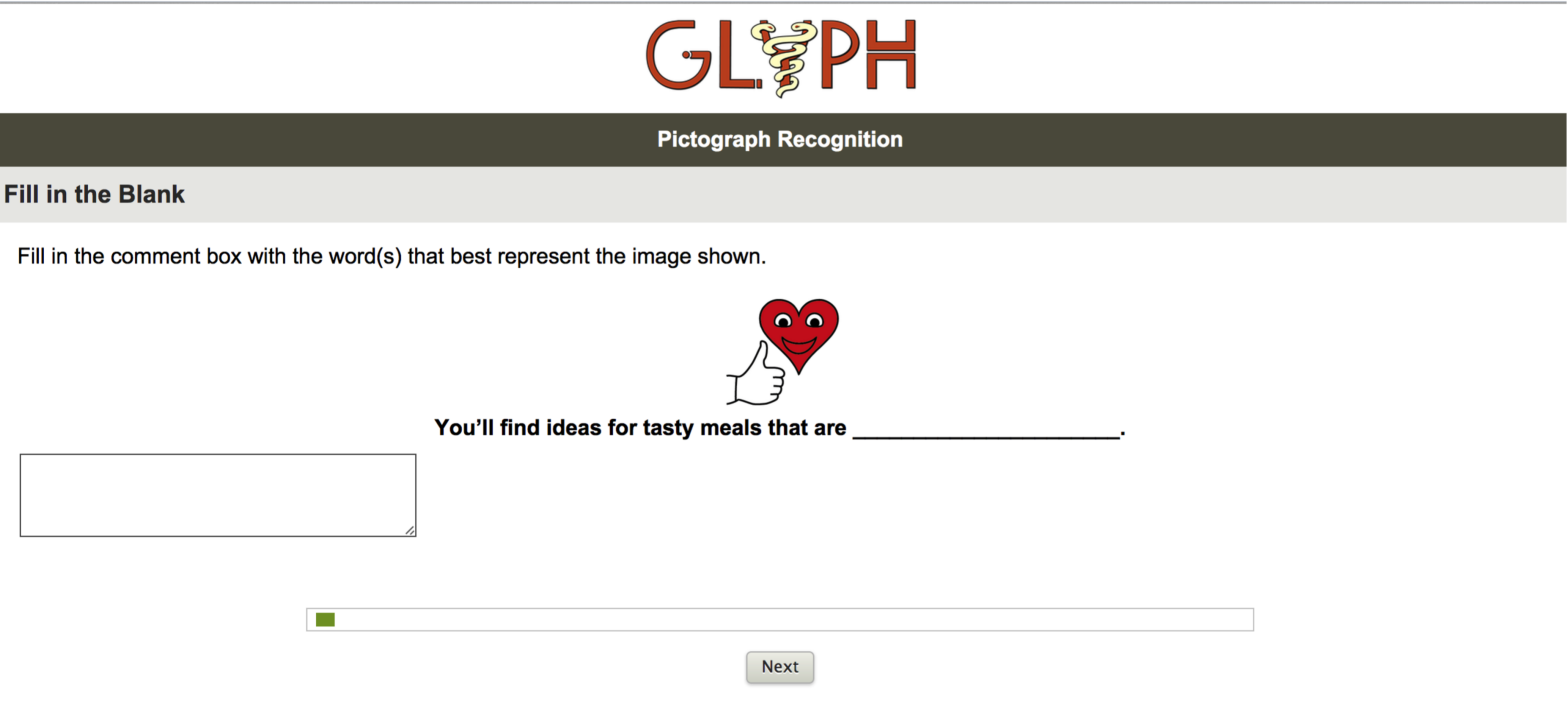
Screenshot of a sample question for both groups.

In the crowdsourcing study, we tested 486 of the 500 images using MTurk. In all, 12 duplicate pictographs associated with different instructions were eliminated to avoid confusion and another 2 pictographs were inadvertently omitted. Similar to the in-person survey, 150 study participants were recruited from MTurk. We requested 15 human intelligence tasks (HITs) per survey, each survey containing up to 50 images to be identified and 7 demographic questions: gender, age bracket, ethnicity, race, education level, first language, and country of residence. Each survey taker received US $6 to complete the survey. We requested that each survey taker be unique and have a “Masters” qualification, which is defined as “consistently completing HITs of a certain type with a high degree of accuracy across a variety of requesters.” Each survey taker was required to answer the questions even if it was a guess. The system prompted the study participants to enter “??” when they could not guess the meaning. The format was “fill-in-the-blank” with a comment box below the sentence (Figure 1).

We used SurveyMonkey [46] as the survey creation tool and for analyzing the responses. Verification that the survey takers were all unique was done based on MTurk user IDs.

In the in-person survey study, the questionnaires with handwritten responses were scanned and answers were transcribed into a database. The MTurk answers were collected using SurveyMonkey and later exported to an Excel spreadsheet. Demographic data were coded for statistical analysis. The questionnaire results were evaluated against the phrases used by the original discharge instructions. The following 4-point Likert scale was used: 1=incorrect, 2=mostly incorrect, 3=mostly correct, and 4=correct. Human reviewers first rated 10% of the questionnaires and an interrater agreement was calculated. Disagreements in rating were resolved through consensus. After interrater agreement reached the conventionally acceptable kappa value of .85, individual reviewers independently rated the remaining questionnaires.

Because each pictograph was tested 15 times and each test result was given a rating from 1 to 4, the sum of the ratings for each pictograph ranged from 15 to 60. In this study, we considered a sum of the ratings less than 40 or a mean rating less than 2.67 as “low” or “ineffective,” indicating a low recognition rate and need for redesign, whereas a sum of the ratings equal to or greater than 40 points (eg, a mean rating equal to or greater than 2.67) was considered as “high” or “effective,” indicating a high recognition rate.

We compared 486 pictographs tested in the crowdsourcing study with their identical counterparts tested in the in-person study. We removed the results in the in-person study that corresponded to the pictographs that were eliminated in the crowdsourcing study due to duplication and omission. We first calculated descriptive statistics for the 2 samples (MTurk and in-person). Mean ratings were then calculated and compared between the in-person hospital sample and online MTurk sample. Afterward, we performed multivariable linear regression analyses to investigate the effects of gender, age, ethnicity, race, education level, and first language on the recognition rates within the 2 populations. Qualitative analyses were then conducted on pictograph characteristics to explore the reasons behind the difference.

## Results

Comparing the in-person and crowdsourcing studies, the 2 recruitment groups differed on several demographic characteristics (Table 1). The MTurk sample had more white and less Hispanic participants, were better educated, and had more native English speakers. We did not limit to US workers only, although more than 93.3% (140/150) of the workers were from the United States. There were 10 non-US workers out of 150 (6.7%), all from Asia. There were 18 Asian participants (18/150, 12.0%) in the in-person group. Asian workers had lower recognition rates than white workers did; however, black or African American, American Indian and Alaska Native, Native Hawaiian and Other Pacific Islander, and other workers scored even lower on average in the in-person study. In our study, the pictograph recognition rate is not a reflection of the participants’ effort because we did not observe any sign of lack of effort by a particular population group. In fact, in the in-person survey, those who were less educated and/or who did not speak English as their first language appeared to spend more time completing the questionnaire.

**Table 1:** Demographics of the in-person and online recruitment groups (N=300).

Demographic characteristics		In-person (n=150)	Online (n=150)	*P*
**Gender, n (%)**				.23
	Male	67 (44.7)	77 (51.7)	
	Female	83 (55.3)	72 (48.3)	
**Age (years), n (%)**				.005
	21-29	46 (30.9)	56 (37.3)	
	30-39	36 (24.2)	44 (29.3)	
	40-49	31 (20.8)	45 (30.0)	
	50-59	22 (14.8)	3 (2.0)	
	60-69	13 (8.7)	2 (1.3)	
	70-79	1 (0.7)	0 (0.0)	
**Race, n (%)**				<.001
	White	86 (57.3)	135 (90.0)	
	Asian	18 (12.0)	10 (6.7)	
	Other	46 (30.7)	5 (3.3)	
**Ethnicity, n (%)**				<.001
	Hispanic	30 (23.1)	4 (2.7)	
	Non-Hispanic	100 (76.9)	144 (97.3)	
**Education (grade), n (%)**				<.001
	≤4	3 (2.0)	0 (0.0)	
	5-8	4 (2.7)	0 (0.0)	
	9-12	25 (16.7)	15 (10.1)	
	>12	118 (78.7)	133 (89.9)	
**First language, n (%)**				<.001
	English	100 (67.1)	139 (92.7)	
	Non-English	49 (32.9)	11 (7.3)	

The mean time spent per survey online was 23.9 minutes (95% CI 22.5-25.3), whereas most in-person participants recruited from the hospital cafeteria area completed the questionnaire in 10-20 minutes. This suggests that online workers were not less attentive.

In the multivariable linear regression model comparing the 2 groups (Table 2), online participants scored significantly higher than the in-person participants after adjusting for demographic characteristics. The majority of pictographs scored well in the recognition test: adjusted mean ratings were 2.95 (95% CI 2.89-3.02) for the in-person sample and 3.14 (95% CI 3.07-3.20) for the MTurk sample on the 4-point Likert scale. The adjusted mean difference was 0.18 (95% CI 0.08-0.28, *P*<.001). This suggests that the MTurk responders were better at recognizing the set of pictographs we tested than the hospital sample were and the difference could not be completely explained by the demographic variables we collected.

**Table 2:** Multivariable linear regression model of mean ratings between the online and in-person groups.

Predictors		Adjusted mean difference (slope)	95% CI	*P*
MTurk		0.18	0.08 to 0.28	<.001
Gender (male)		0.01	−0.07 to 0.10	.75
**Age (years)**				
	21-29	Referent		
	30-39	0.14	0.03 to 0.25	.01
	40-49	−0.09	−0.20 to 0.02	.11
	50-59	0.10	−0.07 to 0.26	.25
	60-69	0.01	−0.20 to 0.22	.92
	70-79	0.37	−0.33 to 1.08	.29
**Race (%)**				
	White	Referent		
	Asian	−0.05	0.24 to 0.14	.59
	Other	−0.17	−0.31 to −0.02	.02
Ethnicity (Hispanic)		0.05	2.79 to 3.08	.54
Education (>12th grade/college)		0.15	0.03 to 0.27	.02
First language (non-English)		−0.54	−0.69 to −0.40	<.001

The model presented in Table 2 identified several predictors of recognition rate in addition to study group. For age, compared with the 21-29 year group, the 30-39 year group mean rating was higher by 0.17 (95% CI 0.03-0.25, *P*=.01). Other older age groups were not significantly associated with rating change. Compared with the white participants, the mean rating for Asian participants was not significantly different (0.05, 95% CI −0.24 to 0.14, *P*=.59) and “other” race ratings were 0.17 higher (95% CI −0.31 to −0.02, *P*=.02). Compared with high-school graduates or lower, college graduates’ mean rating was raised by 0.15 (95% CI 0.03-0.27, *P*=.02). Compared with English as first language, mean ratings for those who did not speak English as a first language ratings were lowered by 0.54 (95% CI −0.69 to −0.40, *P*<.001). No significant differences were detected between mean rating and gender (*P*=.75) or ethnicity (*P*=.54).

**Figure 2:**
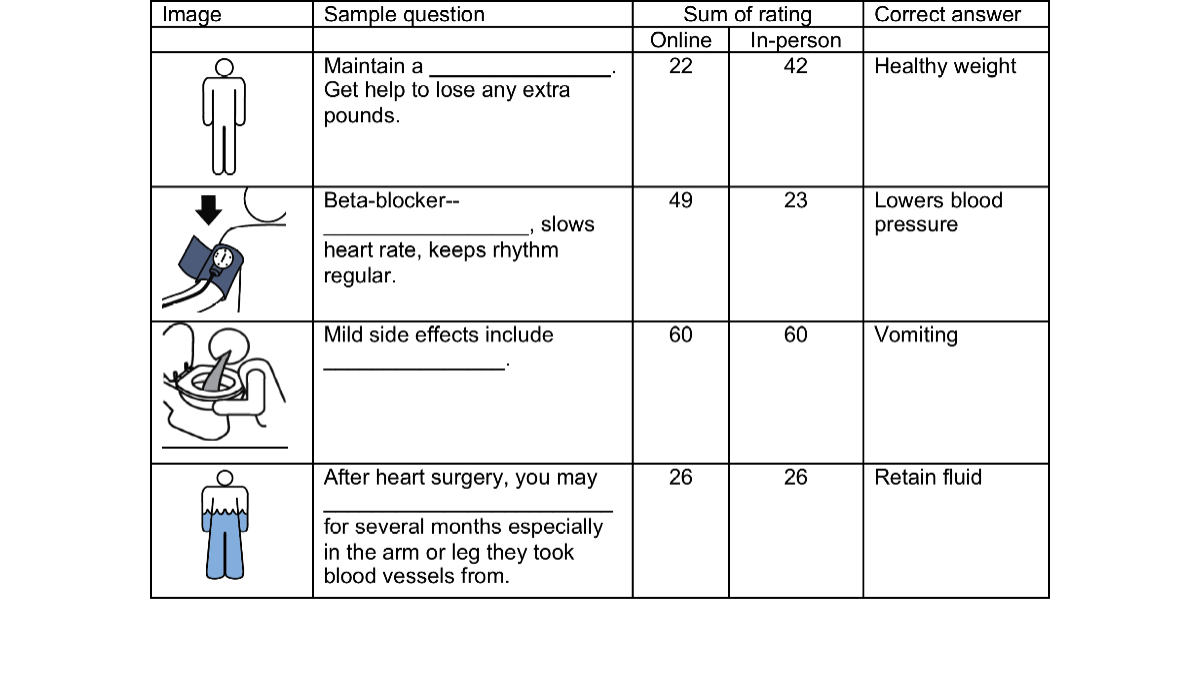
Sample questions with the same or the most different scores from the online and in-person samples.

**Figure 3:**
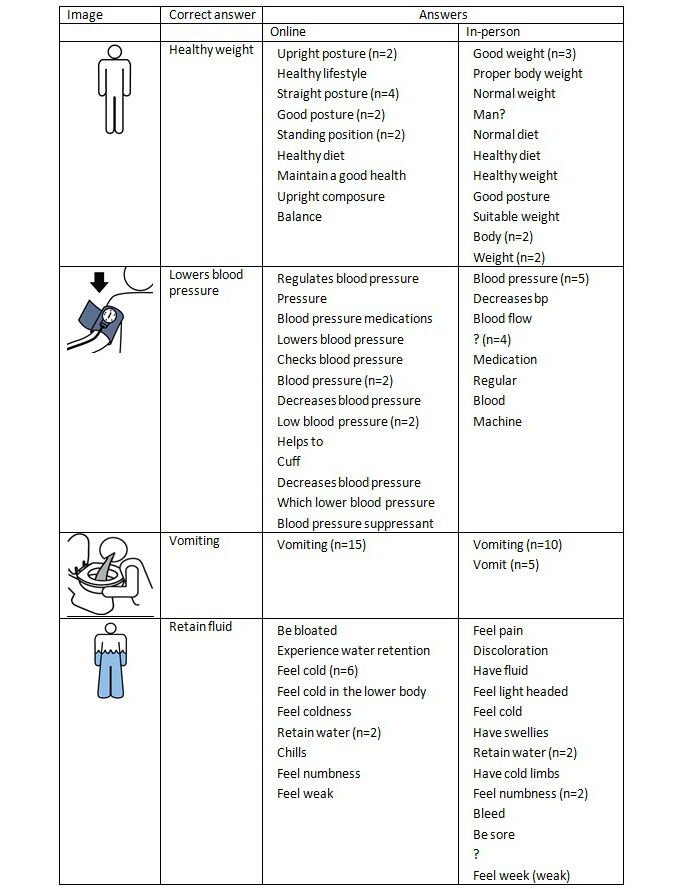
Answers (n=15) to the sample questions from the online and in-person samples.

In the qualitative analysis, we sought to identify general pictographic characteristics that affected recognition by the 2 groups. We examined 3 different categories of pictographs based on recognition ratings. The 3 categories were (1) images that had no variation in mean ratings (n=29), (2) images that scored at least 0.5 points higher in mean ratings with the in-person hospital sample (n=15), and (3) those that scored at least 1 point higher in mean ratings with the online MTurk workers (n=49). Among the 486 pictographs, only 29 had the exact same ratings, although the rating differences were fairly small (<0.5) for the majority of the pictographs. The in-person hospital sample scored higher in 79 images, whereas MTurk workers scored higher in 379 images. Figures 2 and 3 display sample questions and answers with the most similar and the most different scores between the 2 samples.

As part of our analysis, the test pictographs were classified as direct, indirect, and arbitrary according to the representation strategies outlined by Nakamura and Zeng-Treitler [47]. Direct representation explored the visual similarity between a pictograph and its referent, (eg, depicting a thermometer directly). Arbitrary representations were established by social convention (eg, using a red “X” to indicate “no”). Indirect representation explored semantic relations between a pictograph and its referent (eg, using a cactus to represent “dry”). A fourth hybrid category was used for pictographs that contained both indirect and arbitrary elements. Indirect representation was further classified by sematic type. In both samples, the most recognized strategy was direct followed by arbitrary, indirect, and indirect with arbitrary (Table 3). The mean rating within different demographic groups by representation strategy is shown in Table 4. Indirect and indirect with arbitrary strategies were particularly ineffective for older patients, Hispanics, non-Whites, and non-native English speakers. This suggests that the indirect and arbitrary strategies are more culturally dependent.

**Table 3:** Mean rating by representation strategy.

Representation strategy	n (total=486)	Mean rating (SD)	
		Online	In-person
Direct	165	3.45 (0.90)	3.20 (1.08)
Arbitrary	5	3.35 (1.12)	3.09 (1.24)
Indirect	160	3.18 (1.08)	2.77 (1.22)
Indirect with arbitrary	156	3.04 (1.09)	2.56 (1.22)

**Table 4:** The mean rating within different demographic groups by representation strategy.

Demographic groups		Mean rating by representation strategy (SD)				Overall mean rating (SD)
		Indirect	Direct	Indirect with arbitrary	Arbitrary	
**Gender**						
	Male	2.95 (1.18)	3.31 (1.00)	2.77 (1.19)	3.21 (1.16)	3.02 (1.15)
	Female	2.99 (1.16)	3.34 (1.00)	2.82 (1.18)	3.22 (1.23)	3.05 (1.14)
**Age (years)**						
	21-29	3.04 (1.14)	3.33 (0.99)	2.83 (1.18)	3.21 (1.20)	3.06 (1.13)
	30-39	3.04 (1.15)	3.44 (0.92)	2.89 (1.16)	3.36 (1.17)	3.14 (1.10)
	40-49	2.90 (1.20)	3.23 (1.08)	2.74 (1.20)	3.10 (1.20)	2.96 (1.18)
	50-59	2.80 (1.22)	3.30 (1.03)	2.60 (1.23)	3.40 (1.26)	2.92 (1.20)
	60-69	2.83 (1.15)	3.22 (0.98)	2.70 (1.15)	2.80 (1.30)	2.98 (1.10)
	70-79	3.22 (1.20)	3.65 (0.69)	2.25 (1.50)	4.00 (0)	3.46 (0.94)
**Ethnicity**						
	Hispanic	2.67 (1.20)	3.14 (1.10)	2.44 (1.19)	3.54 (0.88)	2.76 (1.20)
	Non-Hispanic	3.03 (1.16)	3.37 (0.97)	2.86 (1.17)	3.26 (1.17)	3.09 (1.12)
**Race**						
	Nonwhite	2.52 (1.21)	3.01 (1.16)	2.34 (1.19)	2.93 (1.25)	2.63 (1.22)
	White	3.13 (1.12)	3.44 (0.92)	2.95 (1.14)	3.28 (1.18)	3.18 (1.08)
**Education**						
	≤12th grade	2.78 (1.17)	3.14 (1.10)	2.48 (1.19)	2.67 (1.34)	2.81 (1.19)
	>12th grade	3.01 (1.17)	3.36 (0.98)	2.85 (1.18)	3.33 (1.13)	3.08 (1.13)
**First language**						
	English	3.12 (1.12)	3.44 (0.92)	2.95 (1.14)	3.32 (1.14)	3.18 (1.08)
	Non-English/other	2.41 (1.21)	2.87 (1.19)	2.22 (1.18)	2.85 (1.26)	2.50 (1.23)

**Figure 4:**
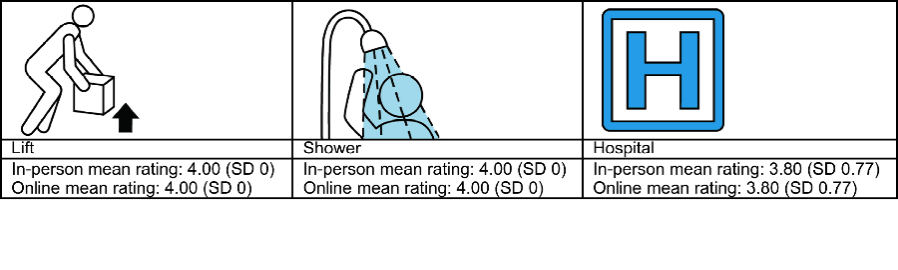
Pictographs that scored the same for both the online and in-person groups.

There were 29 images that received the same recognition scores in both samples. These were generally high-scoring images that represented common objects, activities, behaviors, and common disorders. It should be mentioned there were 3 low scoring images in this category that were not recognizable by either group. This group of pictographs largely represented simple ideas and common behaviors with the use of large fields of open space (Figure 4). Many of these used the direct representation strategy; however, the use of arbitrary symbols was successful in many cases as well.

**Figure 5:**
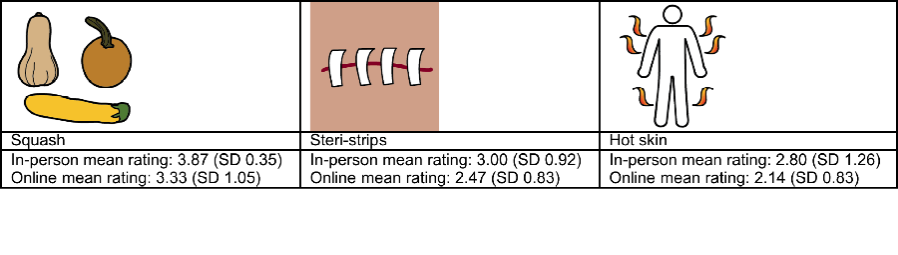
Pictographs that scored higher with the in-person group.

Although the majority of pictographs scored much higher on average with the online group, there were 15 images that scored at least 0.5 points higher with the in-person group. These images tended to have more contrast using color and did not represent overly complex or abstract ideas. With an overall mean rating of 2.95 (SD 0.65) in both groups, these were recognizable in general (Figure 5).

**Figure 6:**
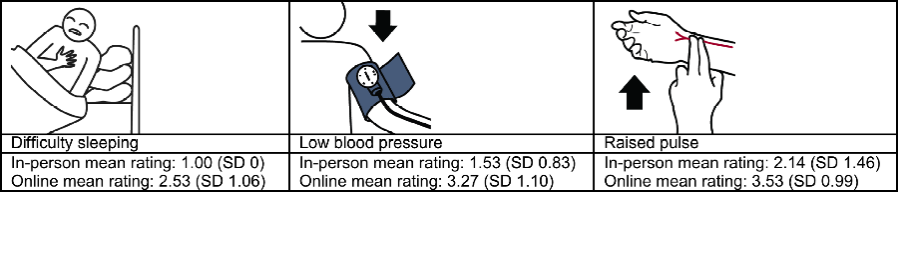
Pictographs that scored higher with the online group.

The final and largest category contained the 49 pictographs that scored at least 1 point or higher by the online group. These images attempted to communicate more complex or abstract concepts than the other 2 categories. The mean rating for the pictographs in this category was 2.77 (SD 0.59) for the in-person sample and 3.31 (SD 0.59) for the online sample. Almost every pictograph in this category used the indirect representation strategy. This category also had many pictographs that contained fine detail and the use of color was not as prevalent as in the other categories (Figure 6). One example is “difficulty sleeping”; although “sleeping” can be easily illustrated, “difficulty” is an abstract and challenging concept to visualize.

These results suggest that the online sample was better at recognizing complex and/or abstract ideas communicated through images. It might be that the MTurks were able to improve their recognition rating by zooming into the screen to see more finely detailed images or they were more familiar with visual icons. Overall, the most efficient way to communicate visually to a diverse audience was the direct representation strategy, employing simple concepts with color and heavy contrast.

## Discussion

This study is the first effort to compare the results of conventional and crowdsourcing recruitment in the health informatics domain. Our crowdsourcing (MTurk) sample had different demographic characteristics from the conventional (hospital patient, visitor, and staff) sample. After adjusting for demographic variables, the crowdsourcing (online) sample scored higher on the pictograph recognition tasks than the conventional (in-person) sample (*P<*.001). This suggests that we cannot simply replace conventional recruitment with crowdsourcing recruitment.

At the same time, the crowdsourcing recruitment was much cheaper and quicker. The data quality was also relatively high. We found no missing data and no transcription was needed. For many pictographs, the differences in the average recognition scores were not dramatic. This suggests that online crowdsourcing is a viable approach for preliminary pictograph evaluation.

Crowdsourcing services—particularly MTurk—have made it easy for scientists to recruit research participants. However, we should not overlook the crucial differences between crowdsourcing and traditional recruitment methods. Not all the tasks that are performed in an in-person setting are suitable for crowdsourcing online. Current general crowdsourcing tools are not specifically tailored for biomedical informatics research. From a human subject researcher’s standpoint, representativeness of a sample is critical. However, tools such as MTurk or SurveyMonkey provide limited capabilities for researchers to sample subjects that mimic the target population of a specific research project. Along the same line, it remains to be explored how crowdsourcing can be incorporated into longitudinal and/or intervention studies.

Creating high-quality and effective pictographs is our goal. To achieve this goal, an iterative process of design and testing was carried out. User testing is intended to identify pictographs that are confusing, allowing those pictographs to be redesigned and retested. In other words, the purpose of the pictograph recognition test is to assess the quality of the pictographs rather than to assess the knowledge and skill of the users. As such, the quality of pictographs being tested will vary and the “wrong” answers are as valuable to us as the “correct” answers.

This study has some limitations. We focused on a single task (pictograph recognition) and a single crowdsourcing service (MTurk). Our conventional sample was recruited from a hospital where our target audience for the pictograph-enhanced instructions receives care. Arguably, a sample recruited from a different location in the United States and a different type of health care facility will have different characteristics and different recognition rates.

In future studies, especially in informatics studies that target patients, we plan to further explore the use of crowdsourcing services. For instance, one of our ongoing projects aims at reducing the disparity in health communication through pictographs. Crowdsourcing is a method that could potentially help us recruit participants from more diverse groups and develop pictographs that are more widely recognizable.

We tested the recognition of health care-related pictographs through crowdsourcing and conventional in-person survey. The self-reported demographics of our online MTurk workers indicated they were younger and more educated than the conventional in-person survey sample. The majority were white and English was their first language. Despite the demographic differences between the 2 study groups, predictors of successful pictograph recognition remain the same: white, college educated, and native English language speaking.

Crowdsourcing has some distinct advantages: it is time-saving, low cost, and less labor intensive (for the researchers). However, our analyses indicated that after adjusting for demographic characteristics, the average pictograph recognition rating of online MTurk and in-person hospital survey participants was significantly different. Therefore, the crowdsourcing approach cannot simply replace conventional survey methods, although it could be used for preliminary studies and quick feedback.

## Abbreviations

HIT: human intelligence task
MTurk: Amazon Mechanical Turk
